# Unlocking Immune Signatures: Surrogate Markers for Assessing VHSV Vaccine Efficacy in Olive Flounder (*Paralichthys olivaceus*)

**DOI:** 10.3390/ani15121728

**Published:** 2025-06-11

**Authors:** Ji-Min Jeong, Mun-Gyeong Kwon, Chan-Il Park

**Affiliations:** 1 Aquatic Disease Control Division, National Fishery Products Quality Management Service (NFQS), Busan 49111, Republic of Korea; 2 Department of Marine Biology and Aquaculture, College of Marine Science, Gyeongsang National University, Tongyeong 53064, Republic of Korea

**Keywords:** viral hemorrhagic septicemia virus, olive flounder, inactivated vaccine, antibody titer, gene expression

## Abstract

*Viral hemorrhagic septicemia virus* (VHSV) is a major pathogen in *Paralichthys olivaceus* (olive flounder) aquaculture, necessitating effective vaccines and evaluation methods. This study assessed immune responses following intraperitoneal administration of an inactivated VHSV vaccine and identified surrogate markers of vaccine efficacy. Fish were sampled weekly over 8 weeks. Antigen-specific antibody titers were measured by ELISA, and the expression of seven immune-related genes (*CD4*, *CD8*, *CD28*, *IgM*, *Mx*, *IFN*, and *IL-1β*) was analyzed in the spleen, kidney, liver, and gill by qPCR. The vaccinated group showed 84% relative survival, while unvaccinated fish experienced 100% mortality. Antibody titers peaked at 4 weeks post-vaccination, aligning with elevated *IgM* expression in the spleen and kidney. *CD4* (kidney) and *CD28* (spleen) expression strongly correlated with antibody titers (r = 0.854 and 0.796, respectively), whereas *IL-1β*, *IFN*, and *Mx* showed moderate associations in specific tissues. These findings indicate that *CD4, CD28*, and *IgM* may serve as molecular indicators of humoral immune responses specifically following inactivated VHSV vaccination in olive flounder (*Paralichthys olivaceus*). Combining gene expression profiling with antibody analysis offers a non-lethal, practical approach to evaluating vaccine efficacy in aquaculture.

## Simple Summary

Viral hemorrhagic septicemia is a deadly viral disease affecting olive flounder, a key species in aquaculture. Although vaccines are available, current evaluation methods rely on artificial infection, which raises animal welfare concerns. This study aimed to establish a scientific and objective method for evaluating vaccine efficacy using immune response markers. Olive flounder were vaccinated with an inactivated virus, and their serum antibody levels and immune-related gene expression in the spleen, kidney, liver, and gills were analyzed. Among the tested genes, *CD4* and *CD28*, involved in immune cell activation, and *IgM*, associated with antibody production, showed strong correlation with antigen-specific antibody titers. These results suggest that measuring the expression of these genes can serve as a reliable and non-lethal approach for assessing vaccine performance. This method supports the development of safer, faster, and more scientifically grounded vaccine evaluation systems, contributing to improved regulatory practices and reduced reliance on lethal challenge tests in aquaculture.

## 1. Introduction

*Viral hemorrhagic septicemia virus* (VHSV), a member of the *Rhabdoviridae* family, is a negative-sense RNA virus with a genome of approximately 11.1 kb, comprising six genes (3′-N-P-M-G-NV-L-5′) [1,2]. Until the mid-1980s, VHSV was restricted to freshwater fish in Western Europe [3], but it was subsequently detected in Pacific salmon [4,5] and has since been reported in more than 60 species of freshwater and marine fish globally [6]. In South Korea, VHSV was first identified in cultured *Paralichthys olivaceus* (olive flounder) in 2001 [7], where it has caused substantial economic losses, particularly during colder seasons.

Due to its high virulence and transmissibility, VHSV is designated as a notifiable infectious disease (Type II) under the Korean Aquatic Animal Disease Control Act, mandating movement restrictions and prohibiting seedling-stage release upon detection. The virus enters the host via oral exposure or gill penetration, subsequently disseminating systemically through the bloodstream and targeting hematopoietic organs such as the kidney and spleen [2]. VHSV can also be excreted in urine, facilitating horizontal transmission via surrounding seawater [8]. Moreover, residual pathogens in aquaculture effluents may pose risks to wild or farmed fish in adjacent waters, with the virus exhibiting environmental persistence in marine systems [9,10].

Currently, no effective treatment exists for VHSV infection, positioning vaccination as the most practical strategy for disease prevention in aquaculture from economic, environmental, and ethical perspectives. Vaccine development requires comprehensive validation for manufacturing quality, safety, and efficacy, typically relying on lethal challenge trials involving vaccination followed by viral exposure and survival monitoring—an approach that necessitates large numbers of experimental animals. In recent years, various types of VHSV vaccines, including monovalent and bacterial combination vaccines, have been developed. However, standardized immune markers for evaluating their effectiveness remain limited. Therefore, there is an increasing need for surrogate indicators that can assess vaccine efficacy without relying on lethal challenge trials.

To reduce animal use and enhance vaccine screening efficiency, alternative non-lethal evaluation methods are critically needed. Gene expression-based immune profiling has emerged as a promising substitute for conventional endpoints such as relative percent survival (RPS) [11]. Non-lethal techniques, including ELISA (Enzyme-Linked Immunosorbent Assay), RT-qPCR, and transcriptomic analyses using blood or mucus, have gained traction as molecular tools for assessing vaccine efficacy [12]. We selected a panel of immune-related genes—*CD4, CD8, CD28, IFN, IL-1β, IgM,* and *Mx*—based on their established functional relevance in teleost immunity. *CD4* and *CD28* are key markers of T helper cell activation and co-stimulatory signaling, while *CD8* is associated with cytotoxic T cell responses. *IFN* and *Mx* play central roles in antiviral defense, *IL-1β* serves as a primary proinflammatory mediator, and *IgM* represents the main antibody isotype in fish.

In this study, we examined the relationship between antigen-specific antibody titers and the expression of immune-related genes in *P. olivaceus* following intraperitoneal administration of an inactivated VHSV vaccine. These findings aim to support the identification of surrogate molecular markers for non-lethal vaccine efficacy assessment in aquaculture species.

## 2. Materials and Methods

### 2.1. Ethics Statement and Experimental Animals

All experimental procedures complied with the relevant Korean animal welfare regulations and were approved by the Institutional Animal Care and Use Committee of the National Fisheries Products Quality Management Service (Approval Code: 2023-4; Approval Date: 28 December 2022). Healthy olive flounder (*Paralichthys olivaceus*, 50 ± 5 g) were obtained from a commercial aquaculture facility with no known history of VHSV outbreaks or immunization practices. All fish were confirmed to be naïve to both VHSV and vaccination prior to use. The fish were acclimated in a recirculating seawater system at 22 ± 0.5 °C for two weeks before experimentation. To ensure the absence of pre-existing infections, kidney and spleen tissues from randomly selected individuals were screened for bacterial and viral pathogens. All fish were humanely euthanized using tricaine methanesulfonate (MS-222; Sigma, St. Louis, MO, USA) before tissue collection.

### 2.2. Virus Culture and Inactivation

The FP-VHS2010-10 strain of viral hemorrhagic septicemia virus (VHSV) was propagated in fathead minnow (FHM) epithelial cells cultured in Dulbecco’s modified Eagle’s medium (DMEM; Gibco, New York, NY, USA) supplemented with 10% (*v*/*v*) fetal bovine serum (FBS) and 1% (*v*/*v*) antibiotic–antimycotic solution (Gibco, New York, NY, USA). Cells were infected with VHSV at a multiplicity of infection (MOI) of 0.01 and incubated at 15 °C. Once complete cytopathic effects (CPE) were observed, the supernatant was harvested by centrifugation (2000 rpm, 10 min, 4 °C) and stored at −80 °C. The virus titer (TCID_50_/mL) was determined in FHM cells using the Reed and Muench method by observing CPE [13].

VHSV (10^8.1^ TCID_50_/mL) was inactivated by adding formalin to achieve a final concentration of 0.2%, followed by continuous magnetic stirring at 4 °C for 24 h. Complete inactivation was confirmed by re-inoculation in FHM cells, which showed no cytopathic effect. The inactivation process was conducted in triplicate to ensure reproducibility.

### 2.3. Vaccination and Sampling

Healthy *P. olivaceus* were randomly assigned to two groups: vaccinated and control. Each group consisted of 50 fish and was maintained in single tanks for sampling purposes. The vaccinated group received an intraperitoneal injection of 100 μL of inactivated VHSV vaccine (concentration: 10^8.1^ TCID_50_/mL), corresponding to a dose of 10⁷ TCID_50_ per fish; the control group received an equal volume of phosphate-buffered saline (PBS).

Blood was collected from the caudal vein at 1, 2, 3, 4, 5, 6, 7, and 8 weeks post-vaccination to assess antigen-specific antibody titers. Sera were separated by centrifugation at 3000× *g* for 30 min at 4 °C, and stored for ELISA. Five fish per group were sampled at each time point (Supplementary Table S1).

For gene expression analysis, spleen, kidney, liver, and gill tissues were collected at the same time points from five fish per group and stored at −80 °C until RNA extraction.

### 2.4. Challenge

Preliminary trials were conducted to determine the optimal VHSV challenge dose. Fish (n = 10 per group) were intraperitoneally injected with VHSV at 10^4^ or 10^5^ TCID_50_/fish, and cumulative mortality was monitored daily. Both doses resulted in 100% mortality; however, the 10^5^ TCID_50_/fish group exhibited rapid mortality within 7 days. Therefore, 10^4^ TCID_50_/fish was selected as the final challenge dose to allow appropriate assessment of vaccine efficacy. In the main trial, 30 olive flounder per group were injected with this dose in three replicates at 3 weeks post-vaccination, and mortality was recorded throughout the observation period.

### 2.5. Antigen-Specific Antibodies

Serum antibody titers were quantified by ELISA. A 96-well microplate (Nunc, Rochester, NY, USA) was coated overnight at 4 °C with 100 μL/well of inactivated VHSV antigen (10⁶ TCID_50_/mL) diluted in 50 mM carbonate buffer (pH 9.6). After washing with 0.05% Tween-20 in PBS (pH 7.2), the wells were blocked with 100 μL of 2% BSA in washing buffer at room temperature for 2 h. Serum samples were diluted 1:10 in PBS containing 0.3% BSA and incubated at 100 μL/well for 1 h. PBS alone served as a negative control. Following washing, 100 μL of mouse anti-flounder IgM antibody (Aquatic Diagnostics, Stirling, UK; 1:5000 dilution in PBS with 0.3% BSA) was added and incubated at 37 °C for 1 h. After another wash, 100 μL of goat anti-mouse IgG conjugated to alkaline phosphatase (Sigma, Livonia, MI, USA; 1:5000 dilution) was added and incubated under identical conditions. For color development, 100 μL of substrate solution (0.1% p-nitrophenyl phosphate [pNPP] in 50 mM carbonate–bicarbonate buffer containing 0.5 mM MgCl_2_, pH 9.8) was added and incubated in the dark at room temperature for 30 min. The reaction was stopped with 50 μL of 2 M NaOH.

Absorbance was measured at 405 nm using an ELISA plate reader (Molecular Devices, San Jose, CA, USA), and OD values were compared between groups.

### 2.6. Gene Expression After Vaccination

Expression levels of immune-related genes were analyzed in spleen, kidney, gill, and liver tissues. Total RNA was extracted using TRIzol Reagent (Invitrogen, Carlsbad, CA, USA), and first-strand cDNA was synthesized using a commercial kit (TaKaRa, Joto-ku, Osaka, Japan). Quantitative real-time PCR (qPCR) was conducted using gene-specific primers and TB Green Premix Ex Taq™ (TaKaRa, Joto-ku, Osaka, Japan) on a Thermal Cycler Dice Real-Time System III (TaKaRa, Joto-ku, Osaka, Japan). The thermal cycling protocol consisted of an initial denaturation at 94 °C for 30 s, followed by 45 cycles of 94 °C for 5 s and 60 °C for 30 s. All reactions were performed in triplicate. Elongation factor 1-alpha (EF-1α) was used as the internal reference gene. Relative expression levels of CD4, CD8, CD28, IgM, Mx, IFN, and IL-1β were calculated using the 2^−ΔΔCt^ method. Primer sequences are listed in Supplementary Table S2.

### 2.7. Statistical Analysis

A significant difference in the survival rate between vaccinated and unvaccinated (control) fish was determined using the log-rank test. All data are expressed as means ± standard deviations (SD). Differences in gene expression among the control and vaccinated groups at different time points (1–8 weeks) were analyzed using one-way analysis of variance (ANOVA), followed by Tukey’s multiple comparison test to evaluate pairwise group differences. Statistical significance was set at * *p* < 0.05 and ** *p* < 0.01. Pearson’s correlation analysis was used to assess relationships between gene expression and antigen-specific antibody titers. All statistical analyses were performed using SPSS software, version 19.0 (IBM Corp., Armonk, NY, USA).

## 3. Results

### 3.1. Artificial Infection Experiment

**Figure 1:**
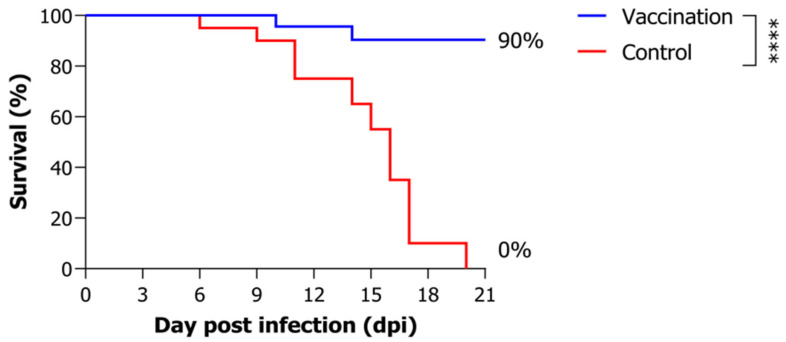
Survival rates of *P. olivaceus* following VHSV challenge. Fish were challenged with VHSV (10^4^ TCID_50_/fish) at 3 weeks post-vaccination. Statistical comparison between groups was performed using the log-rank test (vaccination vs. control, (**** *p* < 0.0001).

Following vaccination with the inactivated VHSV, fish were challenged with VHSV at a dose of 10^4^ TCID_50_/fish. The control group exhibited 0% survival rate by 20 days post-infection, whereas the vaccinated group demonstrated a significantly higher relative survival rate of 90% (Figure 1).

### 3.2. Antigen-Specific Antibodies After Vaccination

**Figure 2:**
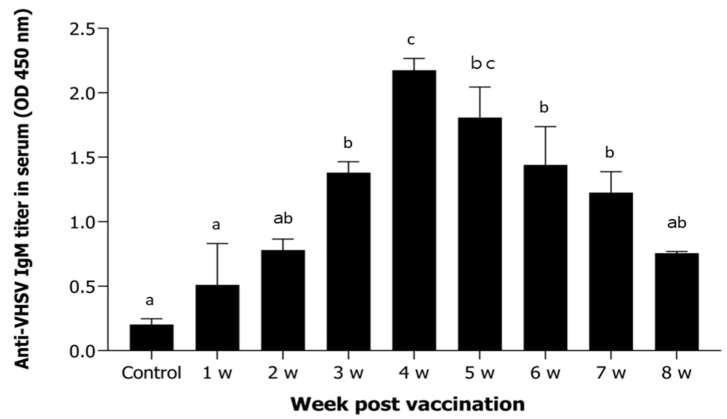
Serum anti-VHSV IgM titers following vaccination. Serum samples were collected weekly over an 8-week period from *P. olivaceus* vaccinated with inactivated VHSV. Anti-VHSV IgM titers were quantified by ELISA at 450 nm. Bars represent the mean ± standard deviation (n = 5). Different letters above the bars indicate statistically significant differences among groups (*p* < 0.05), based on one-way ANOVA followed by Tukey’s multiple comparison test. Groups sharing at least one letter (e.g., ‘ab’) are not significantly different.

Antigen-specific antibody titers increased significantly from 2 weeks post-vaccination and peaked at 4 weeks. Thereafter, a gradual decline was observed through 8 weeks post-administration (Figure 2).

### 3.3. Immune Gene Expression Analysis

Expression levels of immune-related genes (*CD4*, *CD8*, *CD28*, *IgM*, *Mx*, *IFN*, and *IL-1β*) were analyzed in the kidney, spleen, liver, and gill over the 8-week post-vaccination period.

**Figure 3:**
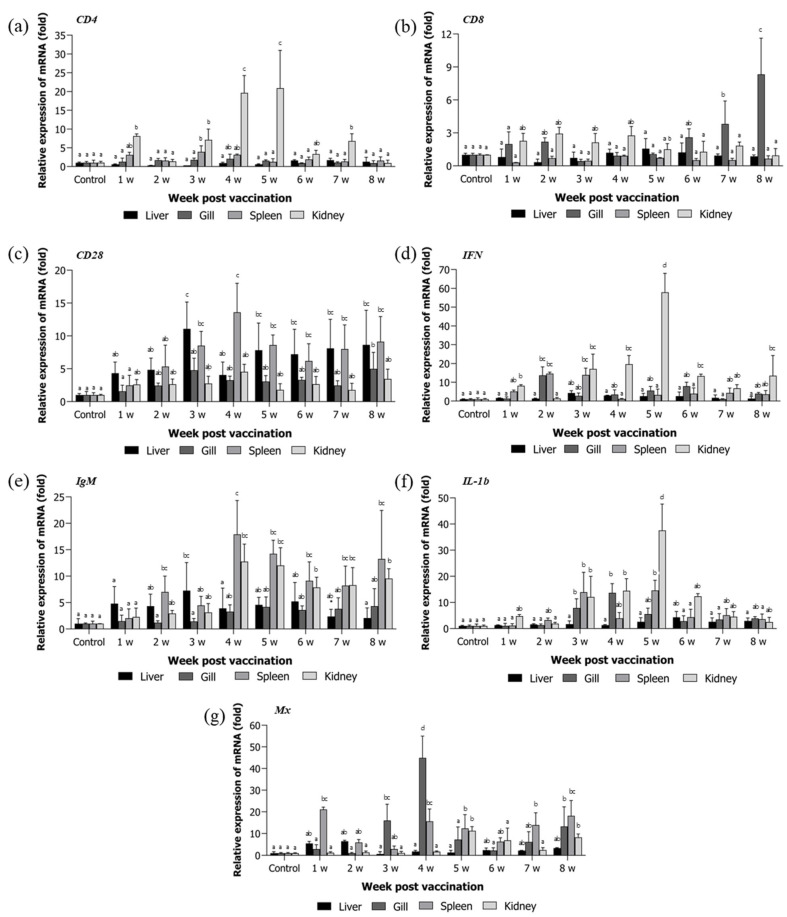
Relative mRNA expression of immune-related genes in four tissues following VHSV vaccination. Transcript levels of *CD4*, *CD8*, *CD28*, *IFN*, *IgM*, *IL-1β*, and *Mx* were measured in the liver, gill, spleen, and kidney of *P. olivaceus* from 1 to 8 weeks post-vaccination. Expression was normalized to *elongation factor 1-alpha* (*EF-1α*) and calculated using the 2^−ΔΔCt^ method. Bars represent the mean ± standard deviation from triplicate biological replicates (n = 3). Different letters above the bars indicate statistically significant differences between groups (*p* < 0.05), as determined by one-way ANOVA followed by Tukey’s multiple comparison test. Groups sharing at least one common letter (e.g., ‘ab’) are not significantly different. (**a**) CD4, (**b**) CD8, (**c**) CD28, (**d**) IFN, (**e**) IgM, (**f**) IL-1β, and (**g**) Mx represent the relative mRNA expression levels of each gene from 1 to 8 weeks following VHSV vaccination.

*CD4* expression was significantly upregulated in the kidney, while changes in the liver, gill, and spleen were minimal throughout the study (Figure 3a). *CD8* expression increased significantly in the gill and kidney from week 1, with a progressive rise in the gill peaking at week 8 (Figure 3b). *CD28* expression was markedly elevated in the liver and spleen, with peak levels in the spleen at week 4 and sustained upregulation at weeks 3, 7, and 8 (Figure 3c). *IFN* expression in the kidney increased from week 1, peaking sharply at week 5 with a > 58-fold elevation compared to controls, followed by a gradual decline (Figure 3d). *IgM* transcription was notably increased in the spleen and kidney, with peaks at weeks 4 and 5. In the spleen, expression rose approximately 18-fold at week 4 (Figure 3e). *IL-1β* expression was significantly elevated in the kidney, spleen, and gill, with a dramatic >37-fold increase in the kidney at week 5 (Figure 3f). *Mx* expression was strongly induced in the gill and spleen; the gill peaked at week 4, while the spleen showed early upregulation from week 1 and maintained high levels through week 4 (Figure 3g).

### 3.4. Correlation Analysis

Pearson correlation analysis was conducted to assess the relationship between antigen-specific antibody titers and the expression of immune-related genes in vaccinated *P. olivaceus*. Significant positive correlations were observed for *CD28*, *IgM*, and *IL-1β*, particularly in the spleen and kidney, indicating their potential as surrogate markers of vaccine-induced immunity.

**Figure 4:**
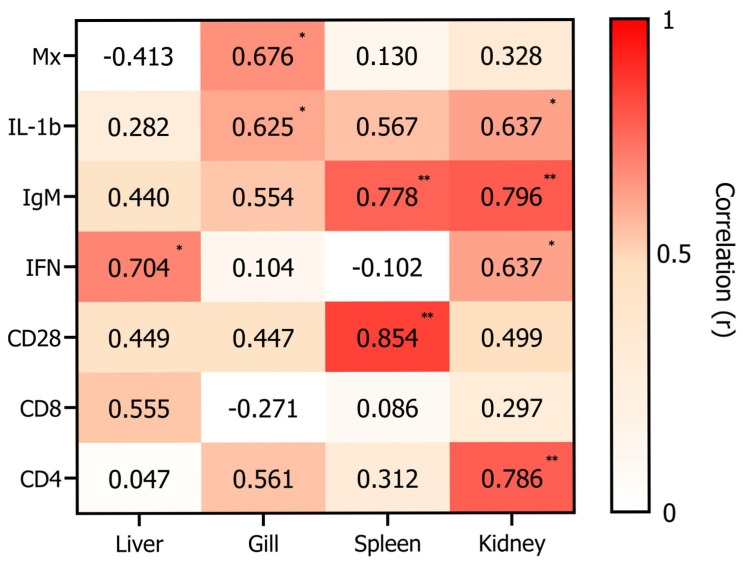
Correlation between serum antibody titers and immune gene expression. Pearson correlation analysis was used to assess associations between VHSV-specific serum antibody titers and mRNA expression of immune-related genes in the liver, gill, spleen, and kidney of *P. olivaceus* following vaccination. Correlation coefficients (r) and *p*-values were calculated using SPSS (version 19.0), with statistical significance defined as * *p* < 0.05 and ** *p* < 0.01. The heatmap illustrates the strength of correlation, with deeper red indicating stronger positive associations.

Additionally, *Mx* expression in the gill and *IFN* expression in the liver and kidney were also significantly correlated with antibody levels. Among all genes analyzed, the strongest correlations were observed for *CD28* in the spleen (r = 0.854, *p* = 0.002) and *IgM* in the kidney (r = 0.796, *p* = 0.005) (Figure 4).

## 4. Discussion

In this study, an inactivated VHSV vaccine was administered intraperitoneally to olive flounder (*P. olivaceus*), and subsequent immune responses were evaluated by measuring antigen-specific antibody titers and the expression of immune-related genes across multiple tissues.

Analysis of antibody titers revealed a peak in antigen-specific responses at 4 weeks post-vaccination, consistent with previous reports on VHSV vaccination [14,15,16]. In the challenge trial, the vaccinated group achieved an RPS of approximately 80%, compared to complete mortality in the control group. These findings suggest that the inactivated VHSV vaccine successfully elicited a robust secondary immune response in *P. olivaceus*.

The innate immune genes *IFN*, *Mx*, and *IL-1β* showed moderate but statistically significant correlations with antigen-specific antibody titers in select tissues. *IFN* and *Mx*, key components of antiviral signaling pathways, were upregulated following vaccination, consistent with prior findings in *P. olivaceus* [17]. *IL-1β*, a proinflammatory cytokine, may contribute to early immune priming by recruiting and activating immune cells [18]. Although these genes were not the most strongly correlated, their early activation post-vaccination supports their potential as auxiliary markers of vaccine responsiveness [19].

In contrast, the adaptive immune-related genes *CD4* and *CD28* exhibited strong positive correlations with antibody titers, emphasizing the central role of helper T cell activation in vaccine-induced immunity in olive flounder. *CD4⁺* T cells orchestrate adaptive immune responses by facilitating B cell activation and promoting antigen-specific antibody production [20]. Elevated *CD4* expression in the kidney likely reflects systemic T cell activation, supporting humoral immunity development [21].

Similarly, *CD28*, a co-stimulatory molecule required for full T cell activation and survival, was highly expressed in the spleen, a major lymphoid organ. This suggests its involvement in antigen-specific T cell activation and long-term immune memory formation. *CD28* signaling has been shown to enhance T cell proliferation and cytokine production in vaccinated flounder, reinforcing its functional role in protective immunity [22]. Notably, previous studies in teleosts have demonstrated functional links between T cell markers and humoral immune responses. For example, *CD28* and *CTLA4*, co-stimulatory molecules expressed on T cells, were shown to enhance T cell activation and indirectly modulate B cell-mediated antibody production in vaccinated European sea bass [23]. Likewise, simultaneous upregulation of *CD4* and *IgM* has been observed in vaccinated fish, suggesting a mechanistic interplay between helper T cell signaling and B cell activation [24]. These observations indicate that tissue-specific expression of *CD4* and *CD28* reflects complementary aspects of T cell-mediated immunity and supports their use as surrogate markers for evaluating vaccine efficacy in teleosts.

Similar approaches have been validated in other species as well. In rainbow trout (*Oncorhynchus mykiss*) vaccinated against *Yersinia ruckeri*, expression levels of T cell-related genes and anti-inflammatory cytokines were used as immune markers correlating with protective efficacy, highlighting their potential for early immunological readouts [25]. In the case of DNA vaccination against infectious hematopoietic necrosis virus (IHNV), interferon-inducible genes such as Mx served as key antiviral indicators, with elevated expression correlating with enhanced protection [26]. Furthermore, in Atlantic salmon (*Salmo salar*), expression of proinflammatory genes like *IL-17A* and acute-phase markers has been used to monitor vaccine-induced reactivity and safety, providing valuable insights into immunogenicity–tolerability balance [27]. These examples across fish species strengthen the utility of immune-related gene markers as non-lethal, predictive tools for vaccine efficacy evaluation.

Consistent with our findings, similar associations between immune gene expression and antibody responses have been reported in other teleost species. For example, Monir et al. demonstrated a strong correlation between *IgM* gene expression and serum antibody levels in red tilapia following bivalent oral vaccination [28]. Similarly, Lund et al. tracked the early temporal dynamics of *IgM* and T cell gene expression in vaccinated Atlantic salmon, showing alignment with antibody development and reinforcing the utility of molecular markers across fish species [29].

While *CD8⁺* T cells play a critical role in cytotoxic responses during later stages of infection, their involvement is more closely associated with cell-mediated rather than humoral immunity [30,31]. The relatively weak correlation between *CD8* expression and antibody titers observed in this study is consistent with the distinct cytotoxic function of *CD8⁺* T cells, which operate primarily within cell-mediated rather than humoral immune pathways. The temporal fluctuations in the expression of *CD4, CD8*, and *CD28* observed in this study are consistent with prior findings in teleost fish, where T-cell–associated genes exhibit dynamic regulation following antigenic stimulation. These variations likely represent the sequential phases of T-cell activation, immunoregulation, and resolution that occur during the adaptive immune response [22,31].

Among the evaluated genes, *IgM* expression in the spleen and kidney exhibited strong correlations with antibody titers, reaffirming its role as a key marker of humoral immune activation following VHSV vaccination. As the predominant antibody isotype during early adaptive responses, *IgM* expression directly reflects B cell activation and antibody production. *IgM* transcriptional dynamics have been shown to closely mirror antibody titers in vaccinated *Paralichthys olivaceus* [32]. Similar findings have been reported following trivalent or live vaccination, where *IgM* transcription in lymphoid tissues correlated with elevated serum antibody levels and enhanced protection [18,33]. Thus, *IgM* expression in immune-relevant tissues serves as a reliable molecular marker of vaccine-induced humoral immunity in teleost.

Collectively, these results highlight *CD4*, *CD28*, and *IgM* as promising surrogate molecular markers for evaluating the immunogenicity and protective efficacy of VHSV vaccines in *P. olivaceus*. Their consistent associations with antigen-specific antibody titers across key immune tissues support the development of gene expression-based, non-lethal methods for vaccine assessment. This molecular approach aligns with evolving paradigms in vaccine evaluation that favor immunological and genomic alternatives to traditional challenge-based testing [34,35,36]. By demonstrating robust correlations between serological responses and immune gene expression, our findings support the refinement and reduction of animal use in vaccine efficacy studies, advancing the implementation of the 3Rs (Replacement, Reduction, and Refinement) in aquaculture research. Moreover, this study provides a scientific basis for future regulatory advancements, promoting the adoption of alternative testing strategies in the approval and quality control of fish vaccines.

## 5. Conclusions

This study examined the expression of immune-related genes in key tissues of *P. olivaceus* following administration of an inactivated VHSV vaccine and evaluated their correlation with antigen-specific antibody titers. Among the analyzed genes, *CD4* and *CD28*, which are integral to T cell activation, and *IgM*, a primary marker of humoral immunity, exhibited strong positive correlations with antibody levels, particularly in the kidney and spleen. These findings indicate that transcriptional expression of these genes may serve as surrogate molecular markers for assessing vaccine-induced immune responses in teleost fish. Additionally, the tissue-specific activation patterns of *IFN*, *IL-1β*, and *Mx* suggest their potential utility as early indicators of innate immune activation, complementing adaptive immune markers. Collectively, this gene expression-based strategy offers a non-lethal, efficient approach for evaluating vaccine efficacy and supports the development of molecular tools to enhance immunization programs in aquaculture.

## Data Availability

Data will be made available upon reasonable request.

## Supplementary Materials

The following supporting information can be downloaded at: https://www.mdpi.com/article/10.3390/ani15121728/s1, Table S1. Experimental design and sampling details for vaccinated and control groups. Table S2. Primer sequences used for qPCR analysis of immune-related genes in olive flounder. Reference [37] are cited in the supplementary materials.

## Abbreviations

The following abbreviations are used in this manuscript:

CD4	cluster of differentiation 4
CD8	cluster of differentiation 8
CD28	cluster of differentiation 28
CPE	cytopathic effect
EF-1α	elongation factor 1-alpha
FHM	fathead minnow
IFN	interferon
IgM	immunoglobulin M
IL-1β	interleukin-1 beta
MOI	multiplicity of infection
MS-222	tricaine methanesulfonate (anesthetic)
Mx	myxovirus-resistance protein
NV	non-virion protein (VHSV gene product)
OIE	World Organisation for Animal Health
RPS	relative percent survival
SPSS	Statistical Package for the Social Sciences
TCID_50_	50% tissue-culture infectious dose
VHSV	viral hemorrhagic septicemia virus
