# Supplementary material for: Unlocking Immune Signatures: Surrogate Markers for Assessing VHSV Vaccine Efficacy in Olive Flounder (Paralichthys olivaceus)
Source: Animals (Basel). 2025 Jun 11;15(12):1728. doi: 10.3390/ani15121728 (PMC12189318; doi:10.3390/ani15121728)
Supplement: Supplementary file 1 [file animals-15-01728-s001.zip › animals-3668484-supplementary.pdf]

Unlocking Immune Signatures: Surrogate Markers for Assessing VHSV Vaccine Efficacy in Olive Flounder (*Paralichthys olivaceus*)

Supplementary Material

Table S1. Experimental design and sampling details for vaccinated and control groups.

Parameter	Vaccinated Group	Control Group
Total number of fish	50 fish	
Injection material	100 μL of inactivated VHSV (10^7 $TCID_{50}$ per fish)	100 μL of PBS
Sampling weeks	Weeks 1 to 8 post-vaccination	
Fish sampled per week	5 fish	
Tissues collected	Blood, spleen, kidney, liver, gill	
Sample preservation	Serum for ELISA; tissues stored at −80 °C for RNA extraction	Same as vaccinated group

Table S2. Primer sequences used for qPCR analysis of immune-related genes in olive flounder.

Gene	Sequence (5' -> 3')	Product size (bp)	Reference
CD4 (AB640684.1)	GTGATCCTAACAAAACCCAGGCAG<br>AGCAGGTTCTTCAACTTTGATCTT	82	Jung et al., 2021
CD8 (AB082957.1)	ATGGACCAAAAGTGGATTCAGATG<br>AACATGTGTGTTGTTCTTCATCTG	108	Jung et al., 2021
CD28 (MT019836.1)	TTCCAACGTCTCATGCACTGG<br>TTTTTGCTGTTTGCGCTCCAC	94	Xing et al., 2021
IgM (AB052744.1)	TTCCTGTAGCTGTGCTGCTG<br>GCTTCCAATCCACTCCAGTC	195	Sohn et al., 2025
IFN (AB435094)	TGGTCTGTCTGTCCCTGTG<br>GCTTCCCGTTGAATCTGT	132	Sohn et al., 2025
IL-1beta (AB070835.1)	AAAAGAGCATCACCCACCCTG<br>CTACCTCAACAAAGCCACCCTT	128	Kole et al., 2021
Mx (AB110446.1)	TCATCTGATTTCGCACCTG<br>TGTCACTCAAACCTGCTGTGCTG	159	Kole et al., 2021
EF-1alpha (XM_069516003)	CCTGGACACAGGGACTTCAT<br>CTGGCCGTTCTTGGAGATAC	120	Sohn et al., 2025
